# Medication Safety in Saudi Arabia: Evaluating the Current Situation and Identifying the Areas for Improvement

**DOI:** 10.3390/pharmacy13020050

**Published:** 2025-04-01

**Authors:** Anwar A. Alghamdi

**Affiliations:** 1 Health Information Technology Department, The Applied College, King Abdulaziz University, Jeddah 21589, Saudi Arabia; nloalgamdi7@kau.edu.sa; 2 Pharmacovigilance and Medication Safety Unit, Center of Research Excellence for Drug Research and Pharmaceutical Industries, King Abdulaziz University, Jeddah 21589, Saudi Arabia

**Keywords:** medication safety, medication error, patient care, patient safety, prescription, pharmacovigilance, medication error hazards, preventive interventions

## Abstract

Drug safety is crucial in healthcare, ensuring the secure and effective administration of medications to protect patient welfare. Drug and medication safety is a major concern among Saudi healthcare providers, with numerous studies outlining the incidence of medication errors and the need for enhanced safety standards. This review will examine the existing level of drug-related safety in Saudi Arabia, categorizing the areas for improvement and highlighting concepts to improve safety practices. The overview discusses the history and evolution of pharmaceutical safety procedures, the present regulatory framework, major stakeholders, and the types and origins of prescription errors. It also examines the role of healthcare personnel and the use of technology and patient education in promoting pharmaceutical safety. The data reveal that the rate of pharmaceutical errors in Saudi hospitals is shockingly high, ranging from 13 to 56 per 100 medication orders, highlighting the urgent need for effective medication safety standards. Despite the formation of the Saudi Food and Drug Authority (SFDA) and the National Pharmacovigilance and Drug Safety Centre, issues such as poor understanding among healthcare providers and the need for more effective reporting methods remain a challenge. The evaluation highlights the deficiencies in ongoing education, such as real-world case scenarios and related trainings, inadequate incorporation of skills in assessment methods, and deficiency in standardized protocols for error reporting. To address these gaps, it is proposed to implement structured competency-based training, simulation exercises must be preferred for periodic skill assessments, and a safe reporting culture should be encouraged for the sake of transparency and learning from errors. We recognize the use of technology, such as electronic health records and computerized physician order input systems, as an important technique for improving medication safety. Future directions include creating national guidelines, establishing a centralized pharmaceutical error reporting system, and fostering a safety culture inside healthcare organizations. By addressing these obstacles and capitalizing on the opportunities indicated, we may improve pharmaceutical safety and, ultimately, patient care and outcomes in Saudi Arabia.

## 1. Introduction

Medication safety is a significant concern in healthcare, influencing patient outcomes and overall quality of life. It is recognized as a global issue due to its serious nature [1], particularly for older patients with multiple health conditions and polypharmacy. The World Health Organization (WHO) has identified medication-related harm as a major public health issue, estimating the burden of medication errors (MEs) and practice costs at approximately USD 37.6 to 50 billion in the United States of America and reflecting nearly the same globally each year [2]. Saudi Arabia, like other countries, faces challenges in medication safety, with studies reporting medication error rates ranging for different MEs, such as prescription errors: 52.70%, dispensing errors: 16.74%, documentation errors: 16.65%, and monitoring errors: 13.91%, and the healthcare professionals are aware of the frequency of drug mistakes and the need for improved safety procedures. Improving safe medication use measures involves encouraging a culture of safety within medical facilities, supporting effective communication regarding drug-related problems, and running training courses on identifying, documenting, and reporting errors and related harms. Non-punitive error reporting policies and cutting-edge technologies like automated dispensing systems can help reduce human errors related to medicine delivery. These approaches can contribute to ongoing improvements in patient safety outcomes [3].

Medication safety in Saudi hospitals is crucial for preventing errors, ADRs, and other drug-related problems. However, 13 to 56% of every 100 medicine orders show common pharmaceutical errors. This highlights the need for strong safety procedures to protect patients. Despite the importance of medication safety, only 9% of hospitals have a medication safety officer, and 30% have a drug safety section. This lack of infrastructure requires the establishment of specific roles and departments to monitor drug safety [4]. High-alert medications pose a significant risk in clinical pharmacy settings because of their nature of causing severe patient harm if used incorrectly. High-alert medications, such as anticoagulants, hypoglycemics, opioids, and chemotherapeutic agents, to name a few, require stringent safety protocols and policies, such as double-checking procedures, independent verifications, and pharmacist-led interventions.

Pharmacovigilance is crucial for maintaining medicine safety in Saudi Arabia, with the National Pharmacovigilance and Drug Safety Centre being established in 2009 [5]. However, healthcare professionals and institutions have varying levels of participation, indicating the need for continuous and extensive practice. Research underscores the significance of continuous education for medical personnel, competency assessments emphasizing medication safety, and cultivating a culture that encourages the reporting of MEs and ADRs [6]. It is recommended to promote national patient safety projects and leveraging technologies to reduce MEs.

Saudi Arabia’s healthcare sector has experienced significant changes and growth recently, with the government prioritizing the development of primary, secondary, and tertiary services [7,8]. Historically, the system provided free healthcare at publicly owned facilities run by government-employed managers and practitioners [9]. However, the system is now shifting from a government-sponsored welfare system to one that is market-oriented, employment-based, and insurance-driven. Despite these changes, the healthcare system continues to face challenges, including a centralized focus on secondary and tertiary care, which raises ethical concerns for medical professionals [10]. Additionally, the dependency on foreign workers and the lack of qualified healthcare professionals are significant issues [9,11].

Saudi Vision 2030 aims to streamline the healthcare system to meet the needs of a rapidly growing and aging population. The new Model of Care (MOC) aims to guarantee long-term financing, adequate availability, and consistent quality of services. Studying drug safety is crucial for improving population health outcomes and ensuring accessibility and quality of healthcare services. This vision emphasizes the need for sensible safety procedures and medication management techniques [11,12].

Saudi Vision 2030 focuses on improving the healthcare system by enhancing efficiency, reducing medical errors, and ensuring drug safety for patients [13]. By focusing on medication safety, Saudi Arabia can reduce risks related to drug-related problems, thereby improving quality of patient care. The alignment of Saudi Vision 2030 with the World Health Organization (WHO) emphasizes the importance of researching drug safety, as the WHO prioritizes safe medication use [12,14]. Saudi Arabia can improve its pharmaceutical safety procedures by following WHO recommendations and guidelines, strengthening its healthcare system, and emphasizing patient safety and treatment quality. Thorough training courses on medication management and safety techniques are crucial for achieving these objectives.

This evaluation aims to assess Saudi Arabia’s present state of medicine safety, highlight areas that need work, and suggest ways to improve drug safety policies. We can increase pharmaceutical safety and finally improve patient care and outcomes in Saudi Arabia by tackling the difficulties and seizing the possibilities found. This review aims to provide an overview of Saudi Arabia’s current pharmaceutical safety rules, procedures, and practices, evaluating their development and identifying successes and challenges.

It also aims to identify risks and gaps in existing literature, highlighting specific hazards related to pharmaceutical safety and areas lacking sufficient data or evidence, with the research question of “what are the key challenges, contributing factors and potential strategies required for improving medication safety in Saudi Arabian healthcare settings”. The review also seeks to identify undiscovered or understudied problems that could compromise Saudi Arabia’s drug safety. The recommendations will be based on policies, strategies, or interventions, proposing evidence-based solutions to address vulnerabilities and gaps in pharmaceutical safety practices.

## 2. Methodology

### 2.1. Criteria for Literature Selection

**Figure 1:**
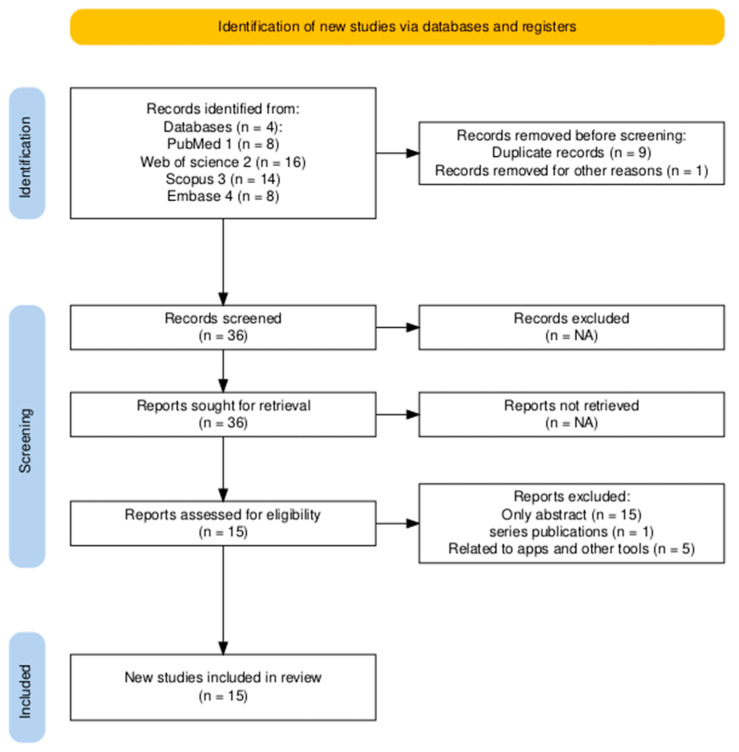
The selection criteria of included studies through PRISMA flow chart.

A comprehensive literature search was conducted employing PubMed, Web of Science, Embase, and Scopus databases to identify required studies on medication safety in Saudi Arabia. The search included peer-reviewed articles published in English between the years 2000 and 2025. The Boolean search strategy was employed in all databases, including the combination of the following terms: medication safety, MEs, pharmaceutical safety along with drug prescribing, preparing, dispensing, administering, and monitoring, add-ons with intervention, prevention, and safety policies related to medications in Saudi Arabia. Duplicated documents were removed, and the studies were screened based on the following inclusion and exclusion criteria. The review followed SANRA (Scale for the Assessment of Narrative Review Articles) and PRISMA (Preferred Reporting Items for Systematic reviews and Meta-Analyses) guidelines (to illustrate the selection criteria) to ensure a transparent and reproducible methodology (Figure 1) [15,16,17].

A total of 46 manuscripts were selected based up on the search performed employing various databases as shown in Figure 1, of which 9 records were identified to be duplicated and one was removed because of irrelevance to the study. Furthermore, only abstracts of 15 studies was available, one was under series publication (duplicated data used) and others were related to the development and application of apps and electronic tools in healthcare. Hence, 15 final manuscripts unique and eligible for inclusion in this review. (Table 1)

Including relevant, high-quality materials for this review, it is crucial to establish the scope of the review manuscript, including research questions or goals. Inclusion criteria include the following: publication type: peer reviewed; quality publications; time span: published in past 25 years; language: preferably English; and relevance to the review problem, i.e., MEs and logistics related to Saudi Arabia [18].

The exclusion criteria include the elimination of low-quality or useless research, such as unpublished work, non-peer-reviewed publications, or significant methodological mistakes. We identify sources from databases and search engines using specific keywords. Screening titles and abstracts is the first step, followed by full-text evaluation of relevant publications for inclusion in the review [18] (Table 1).

**Table 1:** The characteristics of the studies included in the review.

Study	Study Design	Study Characteristics	Interventions	Outcomes	Conclusions
Aljadhey et al. (2013) [4]	National survey	Healthcare providers in Saudi hospitals	Assessment of medication safety practices	Identified gaps in safety protocols and ADR reporting	Need for training and standardized safety guidelines
Tobaiqy & MacLure (2024) [3]	Systematic review	Multiple Saudi hospital settings	Analysis of medication errors	Prescription errors (52.7%) most common; documentation and dispensing errors also significant	Strengthening pharmacovigilance and medication safety initiatives is necessary
Alshammari et al. (2017) [6]	Cross-sectional study	Pharmacists and healthcare workers	Evaluation of pharmacovigilance systems	Underreporting of ADRs and lack of awareness among professionals	Implementation of mandatory ADR reporting and awareness campaigns recommended
Ali et al. (2017) [19]	Retrospective analysis	Large tertiary care hospital in Saudi Arabia	Medication error tracking and classification	High prevalence of prescription and dispensing errors	Need for digital tracking systems and enhanced training programs
Almalki et al. (2011) [8]	Primary care survey	Physicians and nurses in Riyadh	Medication error frequency assessment	High rate of medication errors observed in prescriptions	Systematic medication error reporting and monitoring required
Assiri et al. (2023) [20]	Observational study	Patients receiving long-term medication therapy in family clinics	Identification of preventable ADRs	One-third of ADRs classified as avoidable	Need for better pharmacovigilance and real-time monitoring tools
Alshammari et al. (2016) [21]	Review article	Gulf Cooperation Council (GCC) countries	Overview of pharmacovigilance systems	Variability in ADR reporting and safety policies	Standardizing regional ADR reporting mechanisms necessary
Alharf et al. (2018) [5]	National program evaluation	SFDA initiatives in Saudi Arabia	Assessment of the Saudi Vigilance Program	Reporting systems improving but challenges remain	Strengthening ADR reporting infrastructure needed
Aljeraisy et al. (2011) [22]	Pediatric inpatient study	Pediatric patients in Saudi hospitals	Analysis of medication prescribing errors	Frequent dosing and administration errors	Pediatric-focused medication safety policies required
Khardali et al. (2024) [23]	Nationwide survey	Community pharmacists across Saudi Arabia	Perception and practice of pharmacovigilance	Gaps in knowledge and participation in ADR reporting	Pharmacovigilance training and pharmacist engagement strategies necessary
Egunsola et al. (2021) [24]	Retrospective study	Pediatric patients in Saudi hospitals	Analysis of medication errors	High incidence of pediatric medication errors	Improved safety measures and reporting needed in pediatric care
Al-Aqeel & Alhumaid (2024) [25]	Systematic review	University students in Saudi Arabia	Impact of educational interventions	Positive effect of mentoring programs on medication safety knowledge	Expanding pharmacy education programs recommended
Ahmed et al. (2016) [26]	Systematic review	Hospital settings worldwide	Impact of electronic prescribing	Reduced medication errors in hospitals with e-prescribing	Integration of digital prescription tools in Saudi hospitals suggested
Alhomoud et al. (2024) [27]	Cross-sectional study	Patients with chronic conditions in Saudi Arabia	Medication adherence evaluation	Poor adherence leading to safety risks	Need for pharmacist-led counseling and intervention programs
Alhawassi et al. (2018) [28]	Policy review	National healthcare policies in Saudi Arabia	Assessment of pharmaceutical safety regulations	Limited enforcement of existing medication safety laws	Strengthening regulations and compliance measures needed

ADR: Adverse Drug Reaction; ME: Medication Error; SFDA: Saudi Food and Drug Authority.

### 2.2. Method of Data Extraction

This review article uses a methodical approach for data extraction and analysis to ensure the reliability, correctness, and consistency of the synthesized conclusions. This involves recording relevant information from studies, such as study design, demographic characteristics, interventions, outcomes, and major conclusions. The methodical approach minimizes errors and guarantees recording all significant research features [29]. The review goals then group the data analysis, with qualitative reviews emphasizing thematic synthesis and narrative descriptions and quantitative reviews incorporating statistical assembling. Clear documentation of techniques ensures openness, repeatability, and confidence in the evaluation results [30] (Table 1).

### 2.3. Current Situation of Medication Safety in Saudi Arabia

The Saudi Ministry of Health (MOH) serves as the primary provider and regulator of Saudi Arabia’s primary, secondary, and tertiary healthcare sectors. Other regulatory agencies, such as the Saudi Food and Drug Authority (SFDA), ensure drug safety, efficacy, and quality through strict rules and control. Recent SFDA measures, such as global pharmacovigilance standards and improved monitoring systems, support the regulatory framework. National initiatives like the National Medication Safety Program and the Saudi Patient Safety Centre drive efforts to additional safety culture, apply evidence-based practices, and improve medication safety. These initiatives demonstrate Saudi Arabia’s commitment to patient safety and healthcare system improvement [7].

### 2.4. Literature Review on Medication Errors and Adverse Drug Events Including Population-Specific Data

Medication safety is a crucial aspect of healthcare, ensuring the safe and efficient use of drugs to prevent patient harm. In Saudi Arabia, a few recent studies have identified that MEs related to prescriptions are common, ranging from 13 to 56 per 100 prescription orders. This highlights the need for strong safety measures to protect patients. Only 30% of hospitals have a medication safety section, and 9% have a medication safety officer. In comparison to international benchmarks, such as the USA, where the incidence of ADRs in the case of hospitalized patients was 6.5 to 15 per 100 admissions, and in the case of Australia, where 4% of hospital admission cases are related to medications [4].

This lack of infrastructure necessitates the establishment of specific departments to supervise pharmaceutical safety [4]. Saudi healthcare workers have significant opportunities to enhance pharmaceutical safety, including ongoing education for medical personnel, competency tests focusing on medication safety, and fostering a culture that supports reporting MEs and adverse drug reactions (ADRs).

Saudi Arabian chemists have the responsibility to practice patient counseling, ADR reporting, and drug therapy monitoring to contribute to the safe and efficient use of medications.

Maintaining pharmaceutical safety relies on monitoring and reporting ADRs. In a tertiary healthcare system in Saudi Arabia, immune system problems were the most frequently reported ADRs, with approximately one-third of reported ADRs deemed avoidable or possibly avoidable. This underscores the importance of ongoing pharmacovigilance initiatives and customized prevention actions [31,32].

Various projects and studies have developed Saudi Arabia’s pharmaceutical safety policies. Initially, the focus was on improving basic practices in hospitals, revealing deficiencies. However, in 2009, the Saudi Food and Drug Authority (SFDA) established the National Pharmacovigilance and Drug Safety Centre (Saudi Vigilance) to improve pharmacovigilance efforts and ensure drug safety. This marked a significant turning point in the country’s pharmaceutical safety efforts [33].

**Figure 2:**
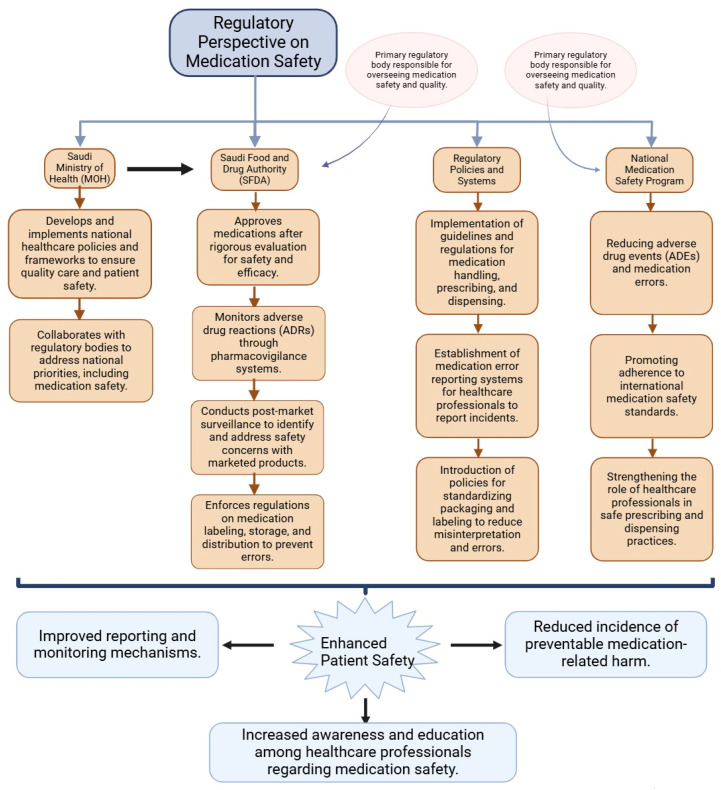
Regulatory Framework for Medication Safety in Saudi Arabia. The Saudi Arabia Ministry of Health (MOH) establishes the main policies for healthcare delivery, and The Saudi Food and Drug Authority (SFDA) is the main body to ensure the safety, efficacy, and quality of medications through pre- and post-market evaluation. The pharmacovigilance system established by the SFDA is responsible for tracking, analyzing, and reporting drug-related problems. The national medication safety program aims to align with the international medication standards to reduce the MEs and to enhance better patient outcomes.

**Figure 3:**
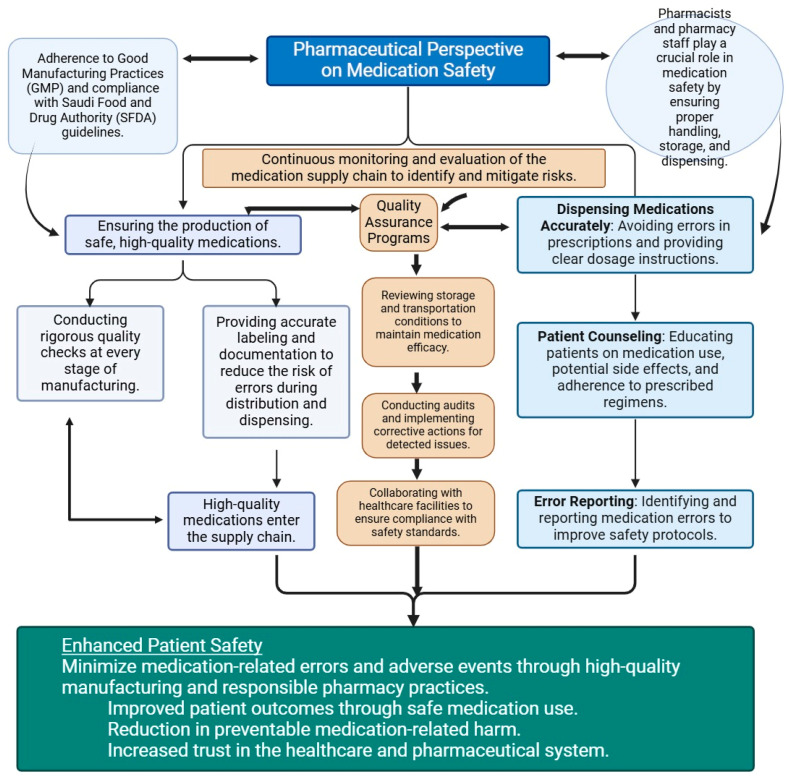
Pharmaceutical perspective on medication safety. Pharmaceutical manufacturers in Saudi Arabia are required to comply with the good manufacturing practices (GMP) to ensure the production of high-quality medications, accurate dispensing, proper labeling, and patient counseling, through which the pharmacists can play a critical role in ensuring medication safety. The entire medication life cycle is monitored by the quality assurance program, and to improve practices in healthcare, continuous professional development programs play a crucial role.

Many healthcare professionals perceived support for pharmacovigilance as relatively new. Studies suggest ongoing education and competency evaluation for healthcare professionals, as well as a culture that welcomes reporting drug-related problems. We suggest technology that can lower errors and support national patient safety campaigns to enhance medication safety procedures (Figure 2 and Figure 3).

Pharmacovigilance is crucial for the guarantee of safe drugs, and while it is relatively new in Saudi Arabia, it is well established in industrialized countries and calls for greater attention from medical professionals and marketing authorization holders. The SFDA and other interested parties have participated in pharmacovigilance events to encourage safe and efficient medication use.

Saudi Arabia’s healthcare system has undergone significant changes in recent years, with the government prioritizing the development of primary, secondary, and tertiary services. Historically, the system provided free healthcare at publicly owned facilities run by government-employed managers and practitioners. However, the system is now transitioning from a government-sponsored welfare system to a market-orientated, employment-based, insurance-driven one. The government’s main concern is the development of Saudi Arabia’s modern healthcare system, which is among the most modern in the Middle East. Despite significant investments in the health industry, the healthcare system still faces challenges, such as a centralized system in major cities, a focus on secondary and tertiary care, and a lack of qualified healthcare professionals. Saudi Vision 2030 aims to modernize the healthcare system to meet the needs of a rapidly growing and aging population, ensuring long-term funding, adequate availability, and consistent quality of service [34].

Various stakeholders and projects regulate drug safety in Saudi Arabia, with the Saudi Food and Drug Authority (SFDA) playing a crucial role in pharmacovigilance. In 2009, the establishment of the National Pharmacovigilance and Medication Safety Centre (Saudi Vigilance) aimed to enhance medication safety and monitor adverse drug events (ADEs). Public health relies on pharmacovigilance, and the SFDA, medical institutions, and professionals are involved in activities. Pharmacovigilance systems address issues like low healthcare practitioner awareness and the need for stronger reporting systems. We recommend practical training and educational interventions to enhance the pharmacovigilance culture. Healthcare experts in Saudi Arabia suggest continuous education, competency testing, and technology to reduce MEs. Pharmaceutical regulations, which prohibit prescription-only medications without a physician’s prescription, require effective implementation and strict enforcement to ensure compliance. The World Health Organization recommends incorporating fundamental applications in healthcare systems to increase medicine safety [35].

Medication safety in Saudi Arabia is a complex issue that involves various stakeholders, including organizations, regulatory agencies, and healthcare providers. The Saudi Food and Drug Administration (SFDA) is the key regulating authority involved in pharmacovigilance activities and ensuring drug safety in Saudi Arabia. The establishment of the National Pharmacovigilance and Drug Safety Centre (Saudi Vigilance) aimed to enhance pharmacovigilance-related efforts. Medical professionals, including doctors, chemists, and other providers, are responsible for reporting ADRs and MEs. Community pharmacists are essential for monitoring drug therapy, patient counseling, and providing pharmaceutical information. Marketing authorization holders are responsible for guaranteeing the safety of their sold products. Academics and policymakers help create legislation and instructional initiatives to enhance pharmaceutical safety procedures [23].

Patients and the general public are also important stakeholders in a complete pharmacovigilance system. Implementing pharmaceutical safety policies and guaranteeing adherence to safety procedures falls to hospital managers and safety committees. Globally, MEs and ADEs pose major issues in healthcare environments. Researchers have conducted research projects to gain a deeper understanding of the frequency, type, and contributing causes of these mistakes in the area [24].

Research projects have examined 10,683 medication error report forms at King Saud Medical City (KSMC) in 2015, as well as four Saudi hospitals [33]. A systematic analysis identified the primary contributing factors to MEs in Middle Eastern countries. Pharmacists at King Faisal Specialist Hospital and Research Centre initiated medication error reports from the pharmacy and verified them under the supervision of the drug safety officer and quality control department. Another study at King Fahd University Hospital in Al Khobar tracked MEs using standard reporting forms by nurses and doctors [3,19,24].

Numerous healthcare environments in Saudi Arabia have categorized and investigated MEs, and the literature has emerged with some important conclusions and classifications. Prescription errors are among the most common types of drug errors, and significant obstacles to reporting drug errors include a lack of knowledge about the reporting policy, workload and time constraints, and the absence of reporting forms [36,37].

a. Prescription Errors:

Incorrect dosages, miscommunication, lack of standardization, inadequate training, and failure to use technology can all contribute to drug errors in healthcare. Incorrect dosages can lead to underdosing or overdosing patients, especially for specific patient populations like pediatrics and geriatrics [38,39,40]. Miscommunication between patients, doctors, and chemists can also lead to misunderstandings, causing potential mishaps [22,34,40]. Variations in prescription forms, abbreviations, and terminology can cause uncertainty among medical personnel, increasing the risk of drug mistakes during dispensing or writing procedures [22]. Insufficient training and education for medical personnel on medication management can also lead to mistakes [22]. The lack of sophisticated technology solutions like electronic prescription systems and drug management software can make it difficult to accurately track prescriptions, increasing the risk of mistakes and degraded patient care [22]. Ignoring possible drug interactions can also lead to negative effects or reduced treatment efficacy. Improving drug safety requires recognizing these challenges, using technology, and promoting a culture of lifelong learning in healthcare environments. This proactive approach not only reduces MEs but also enables medical practitioners to make wise decisions, guided by more efficient and individualized patient care [22,39,40] (Figure 1 and Figure 2).

b. Dispensing Errors:

Mislabeling drugs increases the risk of abuse, as patients may receive incorrect substances or incorrect usage directions. To address this, strong verification procedures and barcode scanning technology can improve medication dispensed accuracy. Establishing a safety culture in healthcare environments promotes honest communication and staff training to identify drug handling hazards [41]. Medication preparation mistakes, such as compounding or repackaging, can pose hazards if not monitored. Regular audits and following set procedures are crucial for minimizing risks. Automated dispensing systems can simplify these procedures, enhancing medication management efficiency and safety [41].

c. Administration Errors:

Incorrect administration techniques can negatively impact treatment and patient outcomes. We must educate and train healthcare workers to stay updated on the latest administration methods, thereby reducing the risk of mistakes. Regular audits and feedback systems are crucial for identifying areas for improvement [42]. Timing errors can also affect medication effectiveness and side effects. Technology like automatic reminders and alerts can help reduce timing mistakes and improve medication management. Comprehensive training courses on timing in medication distribution can strengthen responsibility and improve patient treatment. Combining these techniques can create a more reliable medicine distribution system, foster trust between patients and providers, and ultimately improve health outcomes [42].

### 2.5. Contributing Factors to Medication Errors and Adverse Drug Events

Human factors such as overload, tiredness, and inadequate instruction, as well as environmental factors like poor staffing levels and distractions, can increase the likelihood of prescription mistakes and side effects. Systemic problems in healthcare institutions, such as inadequate team member communication and non-standardized drug administration policies, also contribute to these errors. We should implement a multimodal strategy to address these issues, which includes better training courses, strengthened communication channels, and well-defined procedures [43]. Encouraging a culture of safety and responsibility within healthcare environments can address systemic factors such as poor workflow and communication. Which cultivates trust between patients and healthcare professionals, improving the overall quality of treatment and fostering trust between patients and healthcare professionals. A combined strategy including frequent training courses, feedback systems, and technology integration can simplify procedures and reduce human errors [31]. Technological factors include problems with electronic prescription writing systems, which can threaten patient safety and damage healthcare delivery systems’ efficiency. Addressing these issues is vital, as user-friendly interfaces and strong training programs can reduce the chance of mistakes and improve patient outcomes (Figure 1 and Figure 2). Practical cases of drug mistakes highlight the importance of continuous education and training among medical practitioners, as well as the use of technology to improve communication and simplify drug management procedures. Including best practices and knowledge from these case studies can significantly increase patient safety and promote a culture of responsibility and alertness within medical institutions. Prioritizing these tactics can help healthcare facilities lower the risk of MEs and ensure patient care remains a priority [4,44].

### 2.6. Gaps in the Current Literature and Areas for Further Research

Research on underrepresented patient groups is crucial for developing customized treatment plans and improving health outcomes. We can better understand disease mechanisms and promote fair healthcare policies by addressing these gaps. Recognizing the barriers to clinical study participation can facilitate effective participation and guarantee the inclusion of their viewpoints in the research landscape. Community-based outreach programs and alliances with nearby institutions can close these gaps and result in more inclusive research projects [45]. Insufficient concentrated research on children and geriatric populations reduces the relevance of research results and keeps differences in healthcare outcomes for these vulnerable populations. Collaboration between researchers, doctors, and legislators can help prioritize funds and resources for studies addressing these populations. This cooperative effort creates a better healthcare environment, ensuring all age groups receive the attention and treatment they deserve in medical research and application. Incorporating the perspectives of these age groups into research agendas can create interventions that are successful and culturally and contextually relevant, ultimately improving health outcomes over the lifetime [45].

### 2.7. Lack of Comprehensive Nationwide Studies

We need extensive research to improve our understanding of drug safety and identify potential hazards and advantages for different populations. This will enable healthcare professionals to make informed decisions for patients’ benefit. This research will enhance the evidence base for drug use and support customized medical methods. This will lead to better health outcomes and reduced adverse medication reactions, fostering trust between patients and healthcare systems. Incorporating under-represented groups in studies allows for a more realistic evaluation of drug safety and efficacy across various demographics. This approach can detect potential differences in treatment reactions, ensuring every patient receives the best treatment tailored to their specific situation. This dedication strengthens research validity and helps healthcare professionals make informed decisions [4].

### 2.8. Need for Studies on the Impact of Technological Interventions

There has been a lack of comprehensive research on the effectiveness of e-prescribing and electronic health records in improving patient outcomes, simplifying procedures, and enhancing provider communication. This knowledge will help create a patient-centered healthcare system that reduces treatment access and quality inequalities. By including patient viewpoints and practical applications, research can tailor these developments to address the unique challenges faced by different groups. This approach guarantees the design of technical solutions with practical usability in mind, leading to improved health outcomes across diverse demographics. By fostering a cooperative approach, healthcare environments can provide individualized attention and assistance to each patient, bridging treatment gaps [26,46].

### 2.9. Gaps in Research on the Effectiveness of Current Policies and Practices

This review study assesses current policies and drug safety initiatives to improve patient safety and treatment quality. It suggests best practices to address gaps in the healthcare system, fostering confidence among healthcare practitioners, patients, and legislators. The study highlights the importance of group projects that encourage patient participation in treatment and create a transparent, accountable culture among medical professionals. These projects should include frequent training for practitioners, technology integration for data exchange and communication, and explicit procedures for patient involvement [47].

## 3. Analysis and Discussion

### 3.1. Synthesis of Findings from the Literature

The study shows a constant focus on the value of multidisciplinary approaches, underlining how cooperation within several domains strengthens the validity and usefulness of research results. This trend emphasizes the need for researchers to interact with several points of view and approaches, which ultimately results in more complete answers to difficult challenges. This cooperative approach not only promotes creativity but also helps to share knowledge and resources, therefore opening the path for discoveries that would not be feasible inside separate fields. Such interdisciplinary synergy helps clarify current issues by means of a wider knowledge base and allows researchers to draw on a larger pool of experience and skills [48]. This interdependence creates an atmosphere in which ideas could bloom, thereby advancing science and technology in ways that profoundly affect society. Thus, interdisciplinary cooperation has become indispensable in addressing global issues such as climate change, public health crises, and technological advancements, ensuring that solutions are not only efficient but also sustainable for future generations [49].

### 3.2. Comparison with International Data on Medication Safety

This study highlights the strengths and weaknesses in Saudi Arabia’s healthcare system, underscoring the necessity of incorporating best practices from countries that have successfully enhanced their drug safety policies. By concentrating on these similarities, Saudi Arabia can create focused plans to reduce risks and enhance patient outcomes, thereby promoting a healthcare environment that gives safety and effectiveness in medication management first priority. This commitment to continuous improvement will not only enhance the quality of provided therapy but also foster public trust in the healthcare system, thereby encouraging increased patient participation and adherence to prescribed treatments [50]. Legislators, medical experts, and patients must collaborate to successfully integrate the knowledge from foreign best practices into local systems through these approaches. Such cooperative efforts will make it possible to have strong training programs, uniform policies, and efficient channels of communication—all necessary for advancing medication safety at all levels of treatment. These programs will eventually result in a more educated patient population, equipped to actively participate in their healthcare and therefore lower the likelihood of ADEs and prescription errors [51,52].

### 3.3. Implications for Healthcare Providers and Policymakers in Saudi Arabia

The study highlights the complexity of drug safety in Saudi Arabia, with a high frequency of MEs in hospitals. The lack of dedicated medication safety officers and drug safety units in Saudi hospitals exacerbates this issue. The study also highlights the need for specific departments to supervise drug safety [53]. Pharmacovigilance, which is still emerging in Saudi Arabia, is crucial for ensuring pharmaceutical safety. The Saudi Food and Drug Authority (SFDA) founded the National Pharmacovigilance and Drug Safety Centre (Saudi Vigilance) in 2009 to improve pharmacovigilance operations. However, different healthcare professionals and institutions have varying degrees of participation in pharmacovigilance programs, suggesting the need for more constant and broad involvement [6,53].

Research has identified significant obstacles and opportunities for Saudi Arabia to increase pharmaceutical safety. Essential steps include ongoing education for medical personnel, competency tests emphasizing medication safety, and building a culture that supports reporting drug errors and adverse reactions. We also recommend encouraging and executing national patient safety projects and utilizing technologies that reduce MEs. Overall, enhancing drug safety standards requires a coordinated effort to improve patient safety and promote a safety culture in healthcare environments [54].

### 3.4. Need for Understanding the Scale and Nature of Medication Errors

Medication errors, prevalent worldwide, are a significant concern in Saudi Arabia. For patients, these mistakes might have major medical effects. Several studies have conducted research on the frequency, type, degree, and contributing reasons of drug mistakes in the area. Including Saudi Arabia, a systematic study of the literature on drug mistakes in Middle Eastern nations revealed the frequency and kinds of mistakes, as well as the key contributing causes [36].

In Saudi Arabia, medication mistakes are somewhat frequent in the medical environment. For example, a study at a sizable tertiary teaching hospital in Riyadh looked at the frequency of reporting MEs and elements linked to their underlying causes. The study highlighted significant barriers to reporting drug mistakes, such as inadequate understanding of the reporting policy, heavy workloads, and time constraints [55,56].

Another 2015 study at King Saud Medical City (KSMC) examined 10,683 ME report forms in a retroactive fashion. Indicating that MEs are a major issue in the healthcare environment, this study sought to investigate the occurrence, degree, and reporting of drug mistakes in hospitalized patients [23]. Moreover, chemists started a study at King Faisal Specialist Hospital and Research Centre in Riyadh looking at pharmacy medication error records under assessment by the quality control department and medication safety officer. This retrospective analysis included incorrect drug error reports, highlighting the ongoing issue of MEs in ambulatory care pharmacies [57,58].

### 3.5. Pharmacovigilance: Evolution and Difficulties

Ensuring medication safety and monitoring ADRs depends on the growing field of pharmacovigilance that is developing in Saudi Arabia. Established by the SFDA, the main entity in charge of pharmacovigilance efforts nationwide is the National Pharmacovigilance Centre (NPC). This center connects to the WHO Uppsala Monitoring Centre and reports local Saudi ADRs [23]. Saudi Arabia, like other nations in the region such as Nepal, Sri Lanka, Qatar, Oman, Bahrain, Jordan, Yemen, Lebanon, and Egypt, is still in the process of developing its pharmacovigilance system. Despite this, the NPC has been actively monitoring drug safety and identifying, evaluating, and mitigating ADRs [6].

Complicated ADR reporting forms, inadequate input to healthcare authorities, and nascent risk management strategies are among the primary obstacles in pharmacovigilance in Saudi Arabia. Furthermore, lacking sufficient ADR data might reduce the accuracy of reporting and analysis.

Pharmacists, doctors, and nurses, among other healthcare workers, have different expertise and attitudes toward pharmacovigilance. More than half of the respondents did not know the proper definition of pharmacovigilance, and only a third were aware that the National Pharmacovigilance and Drug Safety Centre is the official body for monitoring ADRs in Saudi Arabia [59]. Despite these challenges, efforts are underway to enhance pharmacovigilance procedures. To encourage methodical reporting of ADRs among healthcare professionals and the general public, the SFDA has, for example, carried out awareness campaigns and training courses [20]. Furthermore, Saudi Arabian community chemists have shown a good attitude towards pharmacovigilance and ADR reporting; many of them regard it as a professional obligation [60].

### 3.6. Key Stakeholders and Their Roles

Medication safety in Saudi Arabia is a complex issue that involves various stakeholders, including organizations, regulatory agencies, and healthcare providers. The National Pharmacovigilance and Drug Safety Centre (Saudi Vigilance) established the Saudi Food and Drug Administration (SFDA) as the key authority in pharmacovigilance activities. Medical professionals, including doctors and chemists, are directly involved in drug administration and patient care, and their reporting of ADRs and medication mistakes is crucial. Community chemists are also essential for monitoring drug therapy, patient counseling, and providing pharmaceutical information [5]. Marketing authorization holders are responsible for ensuring the safety of their products, while academics and policymakers contribute to legislation and educational initiatives. Patients and the general public are also important stakeholders, and their reporting of ADRs and medication mistakes is crucial for a complete pharmacovigilance system. Hospital managers and safety committees are responsible for implementing drug safety policies and ensuring adherence to safety procedures [61,62] (Figure 1 and Figure 2).

### 3.7. Future Directions and Technological Integration

Technological integration is one of the main approaches to raising drug safety in healthcare systems. By automating and standardizing the medication management process, electronic health records (EHRs), computerized physician order entry (CPOE) systems, and barcode medication administration (BCMA) systems can greatly reduce drug mistakes [7].

Notwithstanding the possible advantages, the adoption of these technologies in Saudi Arabia faces various difficulties, including high implementation costs, opposition to change among healthcare workers, and the necessity of thorough training and support [7]. Furthermore, the effectiveness of these technologies is influenced by their appropriate integration into current healthcare processes and the ongoing assessment of their impact on drug safety.

The development of national guidelines and standards for medication safety practices; the creation of a centralized medication error reporting system; and the encouragement of a safety culture inside healthcare institutions constitute future directions for enhancing medication safety in Saudi Arabia [6]. To solve the problems and seize the chances to improve pharmaceutical safety in the nation, cooperative efforts among regulatory authorities, healthcare providers, and other stakeholders are absolutely necessary [6].

Hence, education, technology, and the creation of a strong pharmacovigilance system present many chances for progress even if medication safety presents major difficulties in Saudi Arabia. Successful application of medication safety practices and a decrease in medication mistakes depend on cooperation among important stakeholders and the integration of technology in healthcare systems [21].

### 3.8. Strategies to Reduce Medication Errors and ADEs

#### 3.8.1. Enhancing Education and Training for Healthcare Professionals

Using technology, such as electronic health records, to improve documentation and communication, as well as implementing consistent procedures for medication prescribing and administration, can significantly improve patient safety. These steps can significantly enhance patient safety and ensure the efficient use of drugs, thereby improving health outcomes and reducing healthcare costs. Furthermore, fostering a culture of safety within healthcare institutions encourages staff members to be honest in reporting mistakes, and learning from these mistakes can help prevent such events in the future. Establishing multidisciplinary teams to coordinate medication management can enhance these projects even more by enabling different points of view and knowledge to handle possible obstacles early on. Organizations can ensure that staff members stay up to date with the latest best practices and advancements in medication management by prioritizing continuous education and training for healthcare providers. This dedication to ongoing development not only improves patient treatment but also encourages responsibility and openness inside the healthcare system, building confidence between consumers and doctors [3,63].

#### 3.8.2. Regular Training Programs and Continuous Education

By empowering patients and physicians to have honest conversations regarding medication safety and efficacy, these initiatives can result in a more robust healthcare environment. This collaborative approach fosters a safety culture that values and integrates feedback, thereby decreasing drug errors and improving overall health outcomes. Such projects also open the path for the acceptance of sophisticated technology, such as electronic health records and decision support systems, which further simplify drug management procedures and improve communication among healthcare teams. These technical developments not only improve data exchange but also help healthcare practitioners make wiser judgments, thereby promoting a patient-centered care model that gives safety and quality first priority. This patient-centered approach not only improves the whole healthcare experience but also helps patients participate actively in their treatment, fostering adherence and raising health literacy [64].

#### 3.8.3. Improving Medication Management Systems

It is crucial to lower medication mistakes and guarantee that patients receive the right amounts at the right times, thus minimizing side effects and improving therapeutic results. Combining these systems with electronic health records (EHRs) enables real-time updates and notifications, significantly reducing the likelihood of miscommunication and ensuring all healthcare team members approach patient care in a queue. By means of such linked systems, healthcare practitioners can better share data, therefore enabling more coordinated treatment and finally resulting in better patient outcomes over a variety of demographics. This all-encompassing strategy not only improves patient safety but also enables people to participate actively in their healthcare, therefore encouraging a culture of shared responsibility between patients and professionals [65]. Through the use of technology in this manner, healthcare systems can also spot trends and patterns that guide clinical decision-making, allowing more customized treatment regimens to be catered to the particular requirements of every patient. This shift towards tailored medicine enhances patient involvement and enhances the quality of therapy, as patients feel more engaged in their treatment journey and outcomes. These changes in healthcare delivery are therefore opening the path for creative ideas that give early intervention and preventative treatment top priority, thus improving long-term health results [25].

#### 3.8.4. Adoption of Advanced Technologies Like Barcoding and Electronic Prescribing

By means of technology, this integration simplifies processes and lowers the possibility of mistakes, therefore assuring that patients receive the correct drugs on schedule. Technology enables improved communication among healthcare professionals, which aids in treatment coordination and enhances overall patient safety, thereby reinforcing the importance of technology. This cooperative approach not only improves the effectiveness of healthcare systems but also helps patients to participate actively in their health management, therefore promoting a culture of shared decision-making and responsibility. Healthcare systems can generate a more responsive environment that fits personal needs and preferences by giving patient involvement top priority and using technology. This change to a patient-centered model not only raises satisfaction but also results in better health outcomes since patients who feel informed and active about their treatment are more inclined to follow their plans [27,66].

#### 3.8.5. Strengthening Regulatory Frameworks and Enforcement

It is crucial to implement mechanisms that ensure the regular implementation of these patient-centered approaches in various healthcare environments. This all-encompassing strategy not only improves the quality of treatment but also fosters confidence between patients and doctors, therefore changing the healthcare scene to reflect cooperation and respect. Such a change fosters an environment that values honest communication, facilitates group decision-making, and encourages patients to actively participate in their medical journey. Greater health literacy resulting from this empowerment helps patients to make wise decisions regarding their treatments and lifestyle changes. Healthcare systems can better handle individual requirements and preferences as individuals participate more in their health management, thereby opening the path for compassionate and successful individualized treatment. This change not only enhances patient results but also motivates medical practitioners to use a more all-encompassing strategy, including social, emotional, and physical elements in their treatments. Research has demonstrated that all-encompassing care models enhance patient satisfaction and foster stronger relationships between patients and providers, thereby fortifying the healthcare system and prioritizing well-being over simple treatment [67].

#### 3.8.6. More Vigorous Enforcement of Existing Regulations

Ensuring that healthcare procedures follow set criteria depends on this enforcement; therefore, it guarantees patient safety and fosters system confidence. By increasing responsibility among healthcare providers resulting from strengthened rules, one promotes ongoing industry innovation and progress. These developments not only help patients but also enable medical professionals to provide more efficient and customized treatment, therefore improving the health outcomes over a range of populations. Consistent training courses and seminars can enhance the competencies of medical professionals, ensuring their ongoing awareness of the latest advancements in patient care. Healthcare companies can build an environment that stresses competence and flexibility in the face of changing medical difficulties by encouraging a culture of continuous education and professional growth. This dedication to education not only improves the capacity of individual clinicians but also enriches the whole healthcare system by encouraging teamwork and knowledge-sharing among teams to more successfully address challenging health concerns. This kind of strategy not only raises patient happiness but also promotes creative ideas to solve healthcare inequalities, guaranteeing that every patient, from any background or situation, receives fair and high-quality treatment [68,69].

## 4. Recommendations for Future Research

### 4.1. Longitudinal Studies on Medication Safety

A group of researchers conducted long-term research that tracks advancements and continuous obstacles. [4] These studies can shed important light on the efficacy of drugs over time, guiding clinical recommendations for safer prescription writing and helping to spot possible side effects. Concentrating on various populations in randomized controlled trials improves the knowledge of medication safety and efficacy even more and guarantees that the results apply to many demographics and health issues. Medication safety can also be greatly assessed by broadening the focus of research to include patient-reported outcomes and real-world evidence since it enables the capture of events that may not be clear in conventional clinical studies. Incorporating these methods not only enhances the quality of gathered data, but also empowers patients through their participation in the research process, leading to the creation of more individualized and successful treatment plans. Research technique advancements will foster a more inclusive healthcare environment that values and considers the opinions of every patient during the development of therapeutic interventions. This patient-centered strategy can lead to improved health outcomes by tailoring therapies to meet individual demands and preferences, thereby enhancing adherence and satisfaction with care.

Understanding how these developments change the field of clinical research and open the path for future discoveries in personalized medicine will depend on assessing how technology improves patient involvement and treatment efficacy. It will also be crucial to evaluate possible ethical consequences and guarantee that patient privacy and data security remain first concerns in this fast-changing environment as the integration of technology develops. This multifarious approach not only promotes a patient-centered healthcare system but also stimulates cooperation among stakeholders, including doctors, researchers, and technology developers, to provide solutions that give ethical criteria first priority along with efficacy. Such cooperation can result in the creation of creative tools and platforms that improve patient–provider communication, therefore strengthening adherence to treatment programs and patient outcomes.

Studies have demonstrated the effectiveness of recently developed technology in reducing mistakes. [26] This study underscores the importance of carefully integrating technology into medical procedures, as it has the potential to significantly reduce hazards and enhance overall patient safety. Building on these discoveries, it is vital to keep assessing and modifying these technologies to guarantee they satisfy the changing wants of patients and medical professionals alike. Apart from building confidence between patients and doctors, this continuous evaluation will help promote a transparent and responsible culture within the healthcare sector. Encouragement of honest communication depends on such a society, which can improve patient involvement and empowerment in their own care activities. Empowered patients are more likely to participate actively in their health management, enhancing the results and satisfaction with the treatment received. Healthcare systems may improve this participation even more by giving patient education and assistance top priority, therefore guaranteeing that people have the skills and knowledge they need to make wise decisions about their health. 

By concentrating on underprivileged groups, one can help solve inequalities in healthcare access and results, thereby guaranteeing that everyone receives fair treatment according to their particular requirements. This strategy promotes a cooperative atmosphere where shared decision-making can flourish and helps to build confidence between patients and providers, therefore changing the healthcare experience for all those engaged. As patients take control of their health paths, this metamorphosis can result in more tailored treatment regimens, more respect for medical advice, and more patient empowerment. Such empowerment is essential in promoting a proactive approach to health management, thereby enabling people to actively interact with their healthcare providers and advocate for themselves in the face of obstacles. As people become more aware and involved in their care processes, which results in a better society generally, this proactive involvement can result in improved health outcomes. Maintaining this favorable change in the healthcare sector depends on building a society that values honest communication and cooperation between patients and medical practitioners. 

Focused studies on high-risk populations, including elderly persons, could benefit from better understanding of the ADEs. This study can help to pinpoint particular obstacles that these groups encounter in receiving treatment and support the creation of customized solutions that efficiently meet their particular needs. Such interventions could call for community outreach initiatives, instructional seminars, and technology integration to enable simpler access to healthcare resources. These tactics not only empower underprivileged groups but also help to create a better healthcare system whereby everyone has the chance to obtain high-quality treatment. Encouragement of cooperation between healthcare providers, legislators, and community organizations will help build a strong support system, guaranteeing the long-term viability of these projects. This cooperative method can result in creative ideas addressing structural problems, enhancing health results, and lowering inequalities among many groups. Such developments can also improve the general effectiveness of healthcare delivery, ensuring that resources are directed where they are most needed and that therapies are customized to fit the particular difficulties encountered by different populations.

The pharmacovigilance program of Saudi Arabia has improved a lot over the years; however, key gaps are identified including limited reporting culture, inconsistent integration of pharmacovigilance in healthcare institutions, and insufficient specialized personnel. In comparison to other countries with better established medication safety frameworks—such as in the USA, where the FDA’s MedWatch mandates ADR reporting with an integrated electronic system to monitor medication safety; in the case of the UK, the Yellow Card Scheme promotes transparency and patient involvement in ADR reporting, as well as The National Reporting and Learning System; and Sweden, where the integration of a national database shared across healthcare providers addresses the challenges of ADR reporting—Saudi Arabia faces resolvable issues related to mandatory ADE reporting systems, real-time monitoring the medical data, pharmacovigilance data accessibility, and a lack of reporting due to less awareness. To bridge these gaps, Saudi Arabia should implement mandatory reporting policies for drug-related issues, enhance digital pharmacovigilance tools, and implement real-time safety alerts in hospital systems, as well as adopting public awareness campaigns and implement measures to encourage blame-free cultures as well as incentive-driven reporting for healthcare professionals. These steps could strengthen countries’ medication safety frameworks.

### 4.2. Final Thoughts and Call to Action for Researchers and Policymakers

We should support continuous research and policy creation to improve pharmaceutical safety. Closely working together, researchers and legislators should create an environment that makes patient safety the top priority by means of creative research projects and thorough policy frameworks. Investing in these cooperative initiatives will help us to guarantee that developments in medication safety not only match new issues but also result in better health outcomes for every patient. Engaging stakeholders from many sectors—including healthcare providers, patients, and industry leaders—as we go will help us to develop a unified approach that properly addresses the complexity of medication safety. Along with improving the quality of therapy, this group effort will help communities develop confidence in the safety and effectiveness of their treatments, therefore guaranteeing patient confidence. Building on this foundation, it will be crucial to provide healthcare personnel with continuous education and training to foster best practices and an environment that prioritizes drug safety at all patient care levels. Strong communication plans and feedback systems help stakeholders constantly evaluate the success of their activities and make required changes to raise patient outcomes.

Patients may lack the awareness of reporting MEs, further pharmacovigilance programs never promote patient involvement actively, and patients rely on healthcare professionals for the safety parameters of medication use. Furthermore, cultural barriers may exist which could make patients hesitant to report MEs and related harms, and the blaming culture may not encourage medical professionals to self-report medication-related issues. Limited access to user-friendly ADR reporting platforms, where most systems are designed for the medical professional, but not for the patients further, paper-based or online reporting methods can be complex and time-consuming additions to the already complex ADR reporting system. However, even the institutes do not support the patient-based ADR reporting as they lack awareness and clear policies with limited government involvement in public education campaigns; furthermore, the healthcare providers do not encourage patient reporting, which can easily lead to under reporting [28,69,70,71].

## 5. Conclusions

This review highlights the high prevalence of MEs in Saudi Arabia, with prescription errors (52.70%) being the most common, followed by dispensing (16.74%), documentation (16.65%), and monitoring errors (13.91%). Despite various initiatives, such as the National Pharmacovigilance and Drug Safety Centre, gaps remain in the ME reporting system and pharmacovigilance awareness. The review emphasizes the urgent need for better structured medication safety policy, enhanced training programs, and the integration of technology-driven solutions, such as computerized prescribing and automated medication dispensing.

For healthcare providers, establishing standard error reporting protocols, continuous education, and competency-based assessments are essential to reducing MEs. Policymakers should prioritize mandatory ADR reporting, improve pharmacovigilance infrastructure, and align with the Saudi medication safety framework with international best practices such as the FDA’s MedWatch, the UK’s Yellow Card Scheme, and The National Reporting and Learning System. Pharmacists play a crucial role in medication reconciliation, patient counseling, and ADR monitoring, necessitating enhanced clinical training and interdisciplinary collaboration.

To encourage a non-blaming culture of reporting, healthcare institutions should implement proper error reporting systems, introduce incentive-based ADR reporting, and integrate real-time medication safety alerts within healthcare information systems. Additionally, public awareness campaigns and digitalized pharmacovigilance tools may increase reporting rates and improve patient safety outcomes and the quality of care provided.

### Limitations and Future Directions

Most of the data included in the study were derived from either the regional research or hospital data. Large-scale nation-wide data could prove commendable in deriving a proper understanding regarding the nationwide scenario of medication error reporting standards. Very few data regarding the current policies and their application in medication error reporting were identified, and electronic prescribing and health record integration remain understudied. A limited culture of ADR identification, analysis, and reporting was identified, and many institutions lack a structured method to report the MEs. Limited research publications are identified in the case of vulnerable populations with chronic diseases. Furthermore, data or research publications bearing the comparison between the national and international benchmarks are missing. Limited public awareness, cultural barriers, and a lack of patient-friendly reporting systems contribute to low patient involvement in ME and ADR reporting.

Future research should focus on assessing the impact of current drug safety policies, expanding pharmacovigilance efforts, and exploring innovative strategies to strengthen medication safety frameworks in Saudi Arabia. By addressing these challenges and adopting evidence-based interventions, Saudi Arabia can enhance its pharmaceutical safety system and improve overall patient care outcomes. Furthermore, future studies should focus on evaluating the effect of high-risk medications and their maintenance procedures and policies exploring targeted strategies to minimize their risks in Saudi Arabian healthcare settings.

## Data Availability

All data are contained within the article.
